# Unveiling the recent outbreak of Legionella in Poland: approaches and obstacles – an editorial

**DOI:** 10.1097/MS9.0000000000001434

**Published:** 2023-10-20

**Authors:** Hritvik Jain, Samia Aziz Sulaiman, Prakriti Pokhrel, Noor Ullah Khan, Amogh Verma

**Affiliations:** a All India Institute of Medical Sciences (AIIMS), Jodhpur, India; b Rama Medical College Hospital and Research Centre, Hapur, India; c Kathmandu Medical College and Teaching Hospital, Kathmandu, Nepal; d Health Services Academy, Islamabad, Pakistan; e School of Medicine, University of Jordan, Amman, Jordan

*Dear Editor,*

There has been a recent surge in Legionnaires’ disease (LD) cases in Poland. One hundred sixty-six cases, including 23 deaths, have been reported since 11th September 2023. Earlier on 18th August, public health authorities in Rzeszów, Poland, identified a cluster comprising 158 cases suspected of community-acquired pneumonia; laboratory testing revealed that 15 of those patients were confirmed LD cases. Between 18th August and 11th September 2023, 166 cases of LD were established, and all patients were hospitalized, with 23 associated deaths and a case fatality rate (CFR) of 14%. 23% of cases were reported in Rzeszów county, while the majority, 67% (*n*=112), were reported in the city of Rzeszów, and the remaining 10% (*n*=16) were cases from other locations. Investigations imply that the earliest LD cases first developed symptoms on 30th July, while most contrastingly developed symptoms between August 12th and 16th. All associated deaths (*n*=23) were patients aged between 53 and 98 years with underlying comorbidities. Only hospitalized cases were tested, and since they were principally treated with antibiotics, culture and sequencing testing were not conducted, and cases were thus confirmed for Legionella. Primarily through urine antigen testing or otherwise, by polymerase chain reaction (PCR) respiratory samples (*n*=11)^1,2^.

No new cases have been reported since 7th September; the most recently confirmed cases had an onset date of 29th August. However, investigations have not yet recognized the source of infection and are still ongoing. In addition, to prevent transmission and limit the spread of the disease, cluster investigations and public health activities are currently coordinated by the Polish health authorities^2^. Subsequently, due to the noticeable unusual peak of confirmed cases, associated hospitalizations, and deaths, inconsistent with the Polish annual rate per 100 000 population at 0.1–0.2 per 100 000 population during 2017–2021, considered the lowest amongst European countries, the 2023 Legionellosis outbreak was confirmed^3^.

Studies demonstrate an increase in the global burden of Legionnaires’ disease, which remains an uncommon yet potentially fatal disease. As per the Centers for Disease Control and Prevention (CDC), there has been a consistent increase in the incidence of Legionnaires’ disease since the year 2000, and the actual number of cases may be between 1.8 and 2.7 times greater than the reported figures suggest^4^. The highest annual notification rate to date in the European Union/European Economic Area (EU/EEA) was reported in 2021, 2.4 cases per 100 000 population, a 26.3% rise from the numbers reported in 2020, at 1.9 cases per 100 000 population, despite the slight decrease observed between 2019 and 2020, the earlier at 2.2 cases per 100 000 population. From 2020, a 38% increase in travel-associated Legionnaires’ disease was also demonstrated by the annual report in the EU/EEA^3^. The 2019 CDC study in the United States revealed similar results, indicating that the crude national incidence rate increased significantly, rising from 0.42 cases per 100 000 in 2000 to 2.71 cases per 10 000 in 2019. The highest recorded incidence occurred in 2018, reaching 3.04 cases per 100 000 people. The annual reports for 2018 and 2019 indicated that 15 and 17% of the cases, respectively, were associated with travel^5^.

LD is known to be caused by exposure to the pathogenic gram-negative bacteria genus Legionella; waterborne Legionella pneumophila is considered the most common causative agent for both cases and outbreaks (Fig. 1). The bacteria multiplies in alveolar macrophages intracellularly where subsequent alveolar, destructive inflammation is induced due to the action of bacterial enzymes and recruited neutrophils and monocytes^6^.

**Figure 1:**
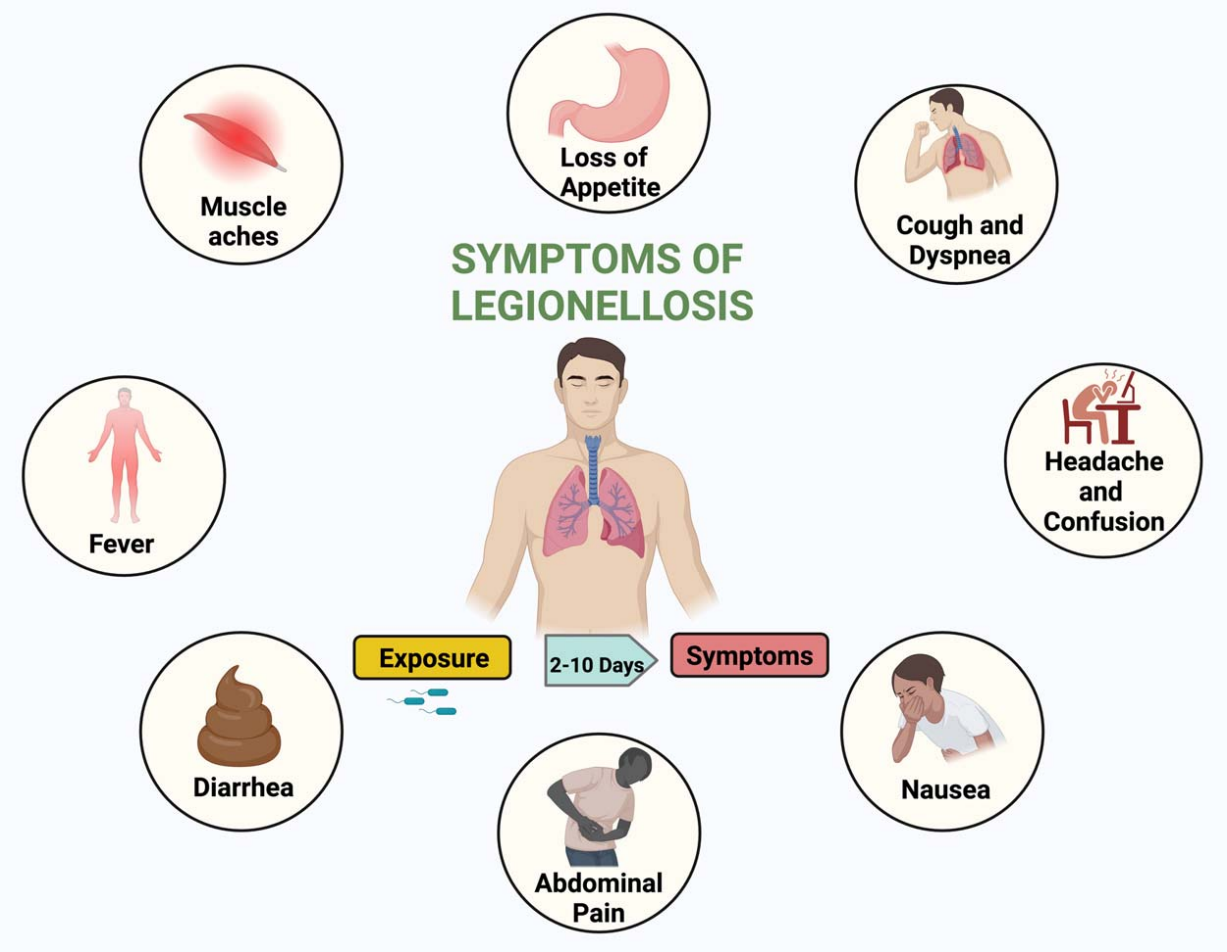
Pathogenesis of Legionnaires’ disease: (1) Sources of infection: Legionella bacilli are attached in biofilms within man-made and natural water systems. (2) Exposure: Human exposure to Legionella bacilli is through inhalation or aspiration to the lungs. (3) Immune response: Legionella intracellularly in alveolar macrophages, causing lung inflammation as a result of recruited immune cells. (4) Infection: Manifestations begin in patients after the incubation period of 5–6 days commonly. Legionellosis most typically presents as acute pneumonia and less commonly as a non-pneumonic type or otherwise, with extrapulmonary manifestations.

LD has a propensity to affect individuals above 50 years, particularly those with any underlying chronic condition or being immunocompromised. Moreover, studies have demonstrated that it is also more common amongst the male population, attributed to differences in behavioral characteristics or occupational exposure patterns. According to a World Health Organization (WHO) report, 75–80% of the patients are above 50, and 60–70% are males^7^. Aquatic and man-made water bodies, such as cooling towers, water heaters, and air conditioners, are ideal for Legionella growth. As a result, epidemics are frequently related to contaminated water supplies. There is also a seasonal preference for the disease, with most cases occurring in the warmer months when the bacteria can reproduce more easily in water systems. In particular, late summer and early fall are the times when legionnaires’ disease epidemics are most frequent^8^.

In 2019, the CDC revised the standardized case definitions of all three categories (Suspected case, Probable case, and Confirmed case) for legionella^9^. The new definitions were based on clinical compatibility with pneumonic-like symptoms and some degree of laboratory evidence to support the diagnosis. Suspected cases were based on clinical compatibility with supportive laboratory evidence for Legionella; probable case definition was reserved for outbreak investigations and required an epidemiologic link during the 14 days before onset of symptoms. A confirmed case was mandated on clinical compatibility with a confirmatory laboratory result. It must be noted, however, that laboratory confirmation for Legionella is considerably less, particularly in developing countries, likely leading to underreporting of cases of the disease.

Legionella presents itself in two forms: the non-pneumonic type and the pneumonic type. The non-pneumonic type (Pontiac sickness) is an acute, self-limiting illness with symptoms similar to flu illness lasting for about 2–5 days. It has an incubation period of a few to up to 48 h. The pneumonic form, however, has an incubation period of 2–10 days, although up to 16 days have been documented in certain outbreaks. The disease is not considered in terms of its latent period since human-to-human transmission is rare and not generally proven. Usually, cases in epidemics are associated with a single contaminated source^10^. Most commonly, Legionella causes acute pneumonia, and less commonly in outbreaks, an influenza-like illness called Pontiac fever, while extrapulmonary disease such as pericarditis and endocarditis is relatively rare^7^.

The Basic Reproductive Rate (R0) ranges from 1 to 5 when there is an outbreak and a contaminated source is the cause of the illnesses. R0 values above 1 indicate the possibility of an outbreak, while R0 values below 1 indicate that the outbreak would eventually end. The primary goal of experts is to bring the R0 number below 1.

The primary method of transmission for legionnaires’ illness is inhaling aerosolized water droplets containing the bacteria. As indicated above, contaminated water sources in artificial systems, such as cooling towers and piping, are the most common causes. Person-to-person transmission is rare, with no documented evidence of direct spread^11^.

There have been outbreaks of LD throughout the world in different areas. Because of inadequate reporting and misdiagnosis, the true global impact is yet to be determined, but the epidemiology is affected by local environmental factors, healthcare infrastructure, and surveillance capabilities. Although more cases are reported in high-income nations with developed healthcare systems, the illness is not just found in these areas. The Melbourne Aquarium (2000), Flint, Michigan (2014–2015), Quincy, Illinois (2015), South Bronx, New York City (2015), Edinburgh, Scotland (2012), and Porto, Portugal (2014) are a few of the relatively more significant outbreaks from the past in addition to the current ones described above^12^. Epidemics have also happened in low- and middle-income nations, frequently linked to poor water infrastructure.

Patients may typically have a 102°F (39°C) or higher fever, chills, and rigors at presentation. Shortness of breath and chest pain upon intake are common respiratory symptoms, along with a persistent cough that may produce fluid or mucus with blood stains (Fig. 2). Some patients have also reported experiencing gastrointestinal symptoms, including nausea, vomiting, and diarrhea. Confusion and altered mental state may appear as the illness worsens, a sign that the central nervous system may be affected^8^. Moreover, the diseases’ complications can be severe, significantly if diagnosis and treatment are delayed past a certain point. Acute respiratory distress syndrome, in which the lungs experience significant inflammation and eventually fail to breathe, is one of the most dangerous sequelae^8^.

**Figure 2:**
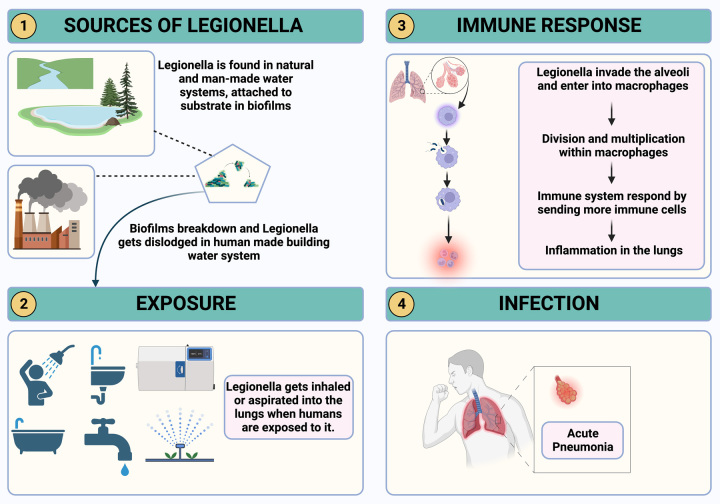
Symptoms and manifestations of Legionnaires’ disease: The majority of symptoms present as respiratory manifestations; nevertheless, systemic symptoms also arise, usually affecting the gastrointestinal system and the central nervous system.

Although there is currently no vaccine for the disease, antibiotics could be administered to treat the sickness itself. Usually, antibiotic therapy is not required for Pontiac fever. For symptomatic LD, the preferred drug is erythromycin, and in severely ill patients, antibiotics are firstly intravenously delivered, and successively, oral therapy could be used. Moreover, rifampin could also be delivered as a second antibiotic in seriously ill patients. It should be noted, though, that early diagnosis remains vital for improving the prognosis and morbidity in this case^6^.

The Polish government has swiftly responded to the Legionella outbreak in Rzeszów, involving daily meetings with crisis staff, water samples, and an epidemiological investigation. Public fountains, water sprays, and communal water sources have been temporarily closed as preventive measures. The Rzeszów Municipal Water and Sewage Company conducted water supply disinfection, health facilities were instructed to inspect their water systems, and public health advice was disseminated to ensure public awareness. This comprehensive response demonstrates a commitment to contain and mitigate the legionella outbreak. The WHO recommends ongoing laboratory analysis, case identification, clinical treatment, contact tracing, outbreak investigation, precautions to prevent new infections, and improvement of infection prevention and control measures. It advises travelers to seek medical assistance and inform their healthcare practitioner of respiratory disease symptoms. The WHO recommends against imposing travel or trade restrictions on Poland^1^.

A thorough strategy is necessary to combat a legionellosis epidemic. Priority must be given to prompt identification and diagnosis by diligent symptom awareness, precise diagnostic testing, and the construction of an effective monitoring system. Early intervention may lessen the severity of the sickness, particularly in situations when an infection is suspected or proven. Environmental studies and water management strategies must be used to find and eliminate possible sources of pollution. In addition to infection control procedures and promoting cleanliness, effective public and healthcare professional communication is essential. Water treatment and routine quality inspection are examples of risk reduction techniques, as is educating building owners. It is crucial for relevant authorities to cooperate and exchange best practices. In order to stop future outbreaks, it is essential to advocate for laws and rules that require regular testing and maintenance of water systems and include preventative measures in building codes.

To minimize their chances of contracting Legionellosis, individuals in the age range of 60–90, who are more vulnerable to the disease, should adopt specific preventive measures. Furthermore, to enhance the management of Legionellosis outbreaks, it is imperative to establish and enforce a standardized protocol for obtaining respiratory secretions or urine samples from suspected cases before administering antibiotics, with a strong focus on healthcare provider training. Additionally, rapid diagnostic tests, such as urinary antigen tests, should be prioritized to swiftly confirm Legionella infection, facilitating timely and appropriate treatment decisions. Healthcare providers should be encouraged to promptly consider Legionella as a potential cause of pneumonia and order the necessary diagnostic tests while promoting antibiotic stewardship principles. This entails advocating for judicious antibiotic use and advising providers to withhold antibiotic treatment until Legionellosis is either confirmed or reasonably ruled out, enhancing diagnostic accuracy and supporting responsible antibiotic prescribing practices.

## Ethical approval

The information provided in the manuscript does not require an ethics application or approval.

## Consent

No patients were sorted. Not applicable.

## Sources of funding

Not applicable.

## Author contribution

H.J.: conceptualization, methodology, supervision, and writing – review and editing; S.A.S.: investigation, writing – original draft, and writing – review and editing; P.P. and N.U.K.: writing – original draft and writing – review and editing; A.V.: writing – original draft, writing – review and editing, and illustrations.

## Conflicts of interest disclosure

There are no conflicts of interest.

## Research registration unique identifying number (UIN)

Not applicable.

## Guarantor

Hritvik Jain.

## Provenance and peer review

Not applicable.

## Data availability statement

Available through the manuscript (with included figures). No other data were generated.

## References

[1] World Health Organization. Legionellosis – Poland; 2023. Accessed 15 September 2023. https://www.who.int/emergencies/disease-outbreak-news/item/2023-DON487

[2] Reuters. WHO says 23 deaths from Legionnaires’ disease reported in Poland; 14 September 2023. Accessed 15 September 2023. https://www.reuters.com/world/europe/who-says-23-legionellosis-linked-deaths-reported-poland-2023-09-14/

[3] ECDC (European Centre for Disease Prevention and Control). Legionnaires’ disease. Annual Epidemiological Report for 2021. Key facts; 2021. Accessed 15 September 2023. https://www.ecdc.europa.eu/sites/default/files/documents/legionnaires-disease-annual-epidemiological-report-2021.pdf

[4] CDC (Centers for Disease Control and Prevention). Legionnaires Disease History and Patterns. Centers for Disease Control and Prevention; 2021. Accessed 15 September 2023. https://www.cdc.gov/legionella/about/history.html

[5] CDC Legionella Surveillance Reports | CDC; 2021. Accessed 15 September 2023. https://www.cdc.gov/legionella/health-depts/surv-reporting/surveillance-reports.html

[6] Winn WC Jr. Legionella. Nih.gov. University of Texas Medical Branch at Galveston; 2016. https://www.ncbi.nlm.nih.gov/books/NBK7619/21413250

[7] WHO. Legionellosis 2022. Accessed 15 September 2023. https://www.who.int/news-room/fact-sheets/detail/legionellosis#:~:text=Legionellosis%20is%20a%20generic%20term,usually%20lasting%202%E2%80%935%20days

[8] Leung YH Lam CK Cheung YY. Epidemiology of Legionnaires’ disease, Hong Kong, China, 2005–2015. Emerg Infect Dis 2020;26:1695–1702. 32687025 10.3201/eid2608.191244 PMC7392469

[9] CSTE (Council of State and Territorial Epidemiologists). Revision to the Case Definition for National Legionellosis Surveillance; 2019. Accessed 15 September 2023. https://cdn.ymaws.com/www.cste.org/resource/resmgr/2019ps/final/19-ID-04_Legionellosis_final.pdf

[10] CDC (Centers for Disease Control and Prevention). Legionella (Legionnaires’ Disease and Pontiac Fever) Clinical Features; 2021. Accessed 15 September 2023. https://www.cdc.gov/legionella/clinicians/clinical-features.html

[11] ECDC (European Centre for Disease Prevention and Control). Surveillance systems overview; 2022. Accessed 15 September 2023. https://www.ecdc.europa.eu/en/publications-data/surveillance-systems-overview-2020

[12] ECDC (European Centre for Disease Prevention and Control). Legionnaires’ disease. Annual Epidemiological Report for 2021; 2023. Accessed 15 September 2023. https://www.ecdc.europa.eu/sites/default/files/documents/leggionnaires-disease-annual-epidemiological-report-2020.pdf

